# *Shigella flexneri* 3a Outer Membrane Protein C Epitope Is Recognized by Human Umbilical Cord Sera and Associated with Protective Activity

**DOI:** 10.1371/journal.pone.0070539

**Published:** 2013-08-05

**Authors:** Anna Jarząb, Danuta Witkowska, Edmund Ziomek, Anna Dąbrowska, Zbigniew Szewczuk, Andrzej Gamian

**Affiliations:** 1 Department of Immunology of Infectious Diseases, Ludwik Hirszfeld Institute of Immunology and Experimental Therapy, Polish Academy of Sciences, Wroclaw, Poland; 2 Department of Animal Products Technology and Quality Management, Wroclaw University of Environmental and Life Sciences, Wroclaw, Poland; 3 Organic Chemistry Department, University of Wroclaw, Wroclaw, Poland; 4 Wroclaw Research Center Center EIT+, Wroclaw, Poland; Queen’s University Belfast, United Kingdom

## Abstract

*Shigella flexneri* 3a is one of the five major strains of the *Shigella* genus responsible for dysentery, especially among children, in regions of high poverty and poor sanitation. The outer membrane proteins (OMP) of this bacterium elicit immunological responses and are considered a prime target for vaccine development. When injected into mice they elicit a protective immunological response against a lethal dose of the pathogen. The OMPs from *S. flexneri* 3a were isolated and resolved by two-dimension-SDS-PAGE. Two 38-kDa spots were of particular interest since in our earlier studies OMPs of such molecular mass were found to interact with umbilical cord sera. These two spots were identified as OmpC by ESI-MS/MS spectrometry. By DNA sequencing, the *ompC* gene from *S. flexneri* 3a was identical to *ompC* from *S. flexneri* 2a [Gene Bank: 24113600]. A 3D model of OmpC was built and used to predict B-cell type (discontinuous) antigenic epitopes. Six epitopes bearing the highest score were selected and the corresponding peptides were synthesized. Only the peptides representing loop V of OmpC reacted strongly with the umbilical cord serum immunoglobulins. To determine which amino acids are essential for the antigenic activity of the epitope, the loop V was scanned with a series of dodecapeptides. The peptide RYDERY was identified as a minimal sequence for the loop V epitope. Truncation at either the C- or N-terminus rendered this peptide inactive. Apart from C-terminal tyrosine, substitution of each of the remaining five amino acids with glycine, led to a precipitous loss of immunological activity. This peptide may serve as a ligand in affinity chromatography of OmpC-specific antibodies and as a component of a vaccine designed to boost human immune defenses against enterobacterial infections.

## Introduction

The *Shigella* genus comprises of Gram-negative facultative human pathogens that are responsible for intestinal infections with symptoms including abdominal cramps, watery or bloody diarrhea and fever. Recent estimates place diarrhea as the third-leading cause of infant mortality worldwide [1]. Those most affected by *Shigella* infections are children under the age of five [2]–[3]. Among *Shigella* species, *S. flexneri* is responsible for the majority of endemic dysentery cases most common in regions of the world where sanitation is poor. Although antibiotic therapy remains a treatment of choice, the emergence of antibiotic-resistant *Shigella* isolates [4] has made prevention strategies, including vaccines, a public health priority. It must be noted that attempts to develop a suitable vaccine for shigellosis have been unsuccessful [5]–[10]. This lack of preventive treatment results from the difficulties to develop a vaccine that meets all essential criteria. It is expected that i) the vaccine will activate the mucosal immune system, ii) immunity will be long-lasting, iii) the vaccine will be safe, inexpensive and easy to store, iv) it will induce minimal, or no side effects, and v) it will be simple to apply. *Shigella* serotypes targeted for vaccine development include *S. flexneri* 2a, 3a, *S. dysenteriae* 1, and *S. sonnei*. Among these, the first three are more prevalent in developing countries, whereas the last serotype appears in regions with advanced sanitation standards [10]–[11].

There are several strategies commonly used to develop a vaccine. To elicit an immunological response, attenuated [12]–[13], or genetically modified strains of the pathogen are being used as the vaccine. Alternatively, the use of outer membrane proteins (OMP) was proposed. The OMPs markedly contribute to the mechanisms of pathogenicity, progression of the infection, and to the development of the inflammatory response [14]–[15]. These proteins are part of the cell envelope of Gram-negative bacteria and participate in maintaining cell integrity, pathogen adaptation to the environment and interaction with the host cells [16]–[17]. Since the OMPs are first to be recognized by the host’s B- and T-cell immune response systems, they make a good target for the development of a cell-free vaccine.

In earlier studies we have described immunogenic and protective properties of the outer membrane proteins isolated from *Shigella*, *Hafnia* and *E. coli* strains [18]–[19]. Both humoral [20] and cellular [21] protective responses were elicited after immunization of mice with these proteins. Immunized mice retained long-lasting protection against lethal doses of both homologous and heterologous strains of the pathogens. Furthermore, it was shown that the OMP-induced immunity against *Shigella* could be transferred passively to non-immunized animals [22]. When non-immunized mice were injected with serum containing anti-OMP antibodies, they became protected against challenge with a homologous strain of the pathogen.

We have recently demonstrated [23] that outer membrane proteins from *Shigella*, *Klebsiella*, *Hafnia* and *Citrobacter* can react with human sera obtained from healthy individuals. This observation is in agreement with the earlier study by Roy et al. [24], which suggested that the 35- to 38-kDa OMPs are major antigens conferring a protective immune response in an animal model of shigellosis. Other studies [9]–[10] have also shown that outer membrane proteins (mainly 38, 34, 23 and 20 kDa) play a key role in eliciting immune responses. The same authors demonstrated that a 34-kDa outer membrane protein from *S. flexneri* 2a is capable of providing significant protection in rabbits challenged with a virulent strain of *S. flexneri* 2a. Recently, the 34-kDa protein was identified as OmpA [25].

In this paper we describe the purification and identification of a 38-kDa OMP from *Shigella flexneri* 3a. We also constructed the 3D model and predicted B-cell type (discontinuous) antigenic epitopes for this OMP and synthesized the corresponding peptides. Finally, we have identified a minimal amino acid sequence required for the antigenic activity of the peptide that represents the active epitope recognized by umbilical cord serum.

## Results

### Protective Properties of OmpC in Homologous System

The results presented in Table 1 illustrate the protective properties of OmpC. OmpC injected intraperitoneally into mice (20 µg per injection) in the absence of adjuvant partially protected animals against a lethal dose of *S. flexneri* 3a. Immunization with OmpC increases protection against a virulent strain of *S. flexneri* 3a in a dose-depended manner. Application of OmpC in formulation with monophosphoryl lipid A (MPL) adjuvant also raises the immune response and protects mice against challenge. Of note, the route of injection plays a crucial role in eliciting the immune response. Mice injected intraperitoneally with OmpC-MPL mixture survived upon challenge with live bacteria, while mice immunized subcutaneously did not.

**Table 1:** Protective effect of mice immunization with OmpC against a challenge with live *S. flexneri* 3a.

OmpC dose [µg/mouse]	Adjuvant	Survival***
1.6	PBS	0 (6)
4.8	PBS	1 (6)
6.4*	PBS	0 (6)
20	PBS	3 (6)
–	PBS	0 (6)
5	MPL	6 (10)
10	MPL	11 (13)
–	MPL	2 (6)
5	MPL	0 (6)**
–	MPL	0 (6)**

* This group received OmpC intraperitoneally in three doses: 1.6 µg, 3.2 µg and 1.6 µg at week intervals.

** These mice received subcutaneous OmpC injection.

PBS – phosphate buffered saline, MPL – monophosphoryl lipid A;

*** Mice were challenged with 0.2 ml of live *S. flexneri* 3a (1.23×10^8^ cells per mouse). The number of animals surviving the challenge is shown. In brackets is the total number of animals in the experimental group.

### Identification of *Shigella flexneri* 3a Outer-membrane Proteins

**Figure 1:**
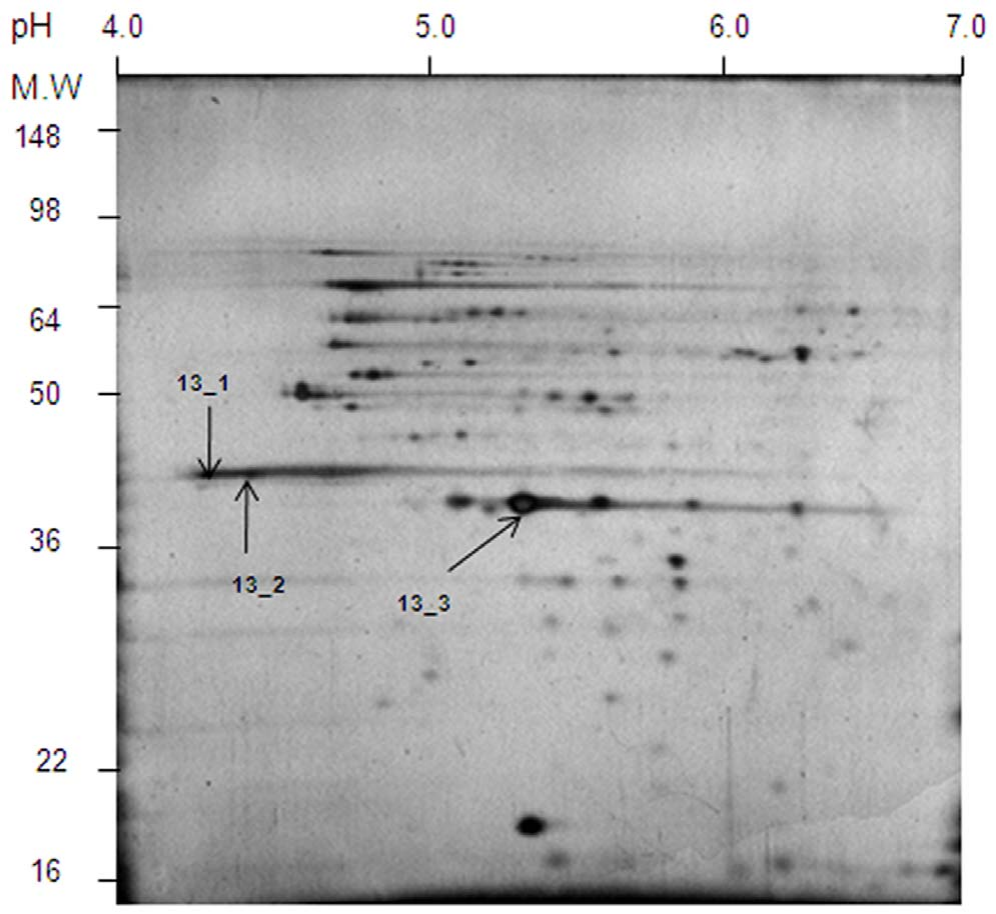
2D-SDS-PAGE of *S. flexneri 3a* outer membrane proteins. 100 µg of crude preparation of the outer membrane proteins was precipitated with TCA. The pellet was washed with 12.5% TCA, acetone and air dried. The precipitate was dissolved in 125 µl of the sample buffer (pH 4–7). The IPG strip (11 cm) was soaked in the sample solution placed in the ceramic container provided with the IPGphor system. The first dimension of the electrophoretic separation was done according to the manufacturer’s recommendations. Proteins resolved on the buffer strip were reduced with DTT and alkylated with iodoacetamide. For the second dimension the IPG strip was placed over a vertical slab of 12.5% polyacrylamide gel prepared according to Laemmli [40]. Protein spots were visualized using the silver staining method as described by Shevchenko et al. [41]. Spots 13_1 and 13_2 were identified as OmpC and spot 13_3 as OmpA (Table 2).

The outer-membrane proteins from *S. flexneri* 3a were extracted with Triton X-100 and were resolved by 2D-SDS-PAGE (Fig. 1). The protein spots distribution pattern on 2D-gel was compared to that described by Ying et al. [26] for *S. flexneri* 2a outer membrane proteins. The distribution of proteins shown in Fig. 1 is very similar to that obtained for *S. flexneri* 2a outer membrane proteins [26]. In the case of *S. flexneri* 2a, two distinctly acidic (pI 4.5–4.6) 38 kDa protein spots were identified as OmpF and OmpC, while the central spot was identified as OmpA [25]. Similar acidic spots (Fig. 1., spots 13_1 and 13_2) were detected on 2D-SDS-PAGE of the OMP preparation from *S. flexneri* 3a. At the center of both gels there is an intense protein spot of pI 5.5 (Fig. 1, spot 13_3). All three spots shown in Fig. 1, two acidic and one central, were extracted from the gel and subjected to in-gel tryptic digest and ESI-MS/MS (electrospray ionization - tandem mass spectrometry) analysis. The obtained MS sequencing data for all three spots are shown in Table 2. Both 13_1 and 13_2 were identified as OmpC, while 13_3 was determined as OmpA. Identification of OmpA at the center of the gel and the overall pattern of the protein spots, make this gel very similar to that obtained for *S. flexneri* 2a outer membrane proteins [26]. Interestingly, no spot corresponding to OmpF was found on 2D-SDS-PAGE. Also, there was no indication of OmpF being present after extensive N-terminal sequencing of the 38-kDa band obtained from one-dimensional SDS-PAGE (data not shown). The only protein found corresponded to OmpC from *S. flexneri* 2a. Differences in the isoelectric point of these two OmpC spots (Fig. 1), could result from post-translational modifications, similar to those described for other prokaryotic proteins [27].

**Table 2:** Identification of selected protein spots extracted from 2D-SDS-PAGE.

Sample ID	NCBI BLASTsearch	Protein name	MW(Da)	Mascotscore *	Identified peptidesequence
13_1	gi|24113600	outer membrane protein 1b (Ib;c)[Shigella flexneri 2a str. 301]OmpC	41377	119	FQDVGSFDYGRGNGFATYRNGSPEGEGMTNNGRTDDQNFGLNRYDERINLLDDNQFTR
13_2	gi|24113600	outer membrane protein 1b (Ib;c)[Shigella flexneri 2a str. 301]OmpC	41377	158	FQDVGSFDYGRGNGFATYRNGSPEGEGMTNNGRTDDQNFGLNRYDERINLLDDNQFTR
13_3	gi|110804972	outer membrane protein 3a[Shigella flexneri 5 str. 8401]OmpA	37402	632	AQGVQLTAKLGYPITDDLDIYTRFGQGEAAPVVAPAPAPEVQTKDGSVVVLGYTDRIGSDAYNQGLSERRAQSVVDYLISKGIPADKISARGMGESNPVTGNTCDNVKAALIDCLAPDRGIKDVVTQPQA

Protein spots were extracted from 2D- polyacrylamide gel with an Eppendorf pipette and approx. 1 mm in diameter gel pieces were destained according to the procedure described by Gharahdaghi et al. [42]. In-gel tryptic digestion of proteins was done using Promega (Madison, WI, USA) Gold Label trypsin, following the manufacturer’s protocol with only minor alterations. The tryptic digest sample (1–10 µl) was injected on a trap column coupled to a C18 Waters NanoEase capillary column 75 µm×50 mm. The ESI-MS/MS experiments were carried out on a QTOF-Global mass spectrometer (Micromass, Manchester, UK).

* Mascot score was obtained after submitting raw data (PKL format) to the Matrix Science website for evaluation (http://www.matrixscience.com/cgi/search_form.pl?FORMVER=2&SEARCH=MIS).

### Sequencing of OmpC from *S. flexneri* 3a

Although protein sequencing by ESI-MS/MS suggested a high degree of homology between OmpC from *S. flexneri* 3a and OmpC from *S. flexneri* 2a, only their DNA sequencing could determine the similarity of these two proteins. The 3′- and 5′- primers for the PCR reaction were designed using a sequence of the *ompC* gene from *S. flexneri* 2a [Gene Bank; 24113600]. The PCR product was sequenced as described in the Experimental Procedures. The sequence determined for *ompC* gene from *S. flexneri* 3a was identical to that of the *ompC* gene from *S. flexneri* 2a.

### Bioinformatic Analysis of the *S. flexneri* 3a OmpC

**Figure 2:**
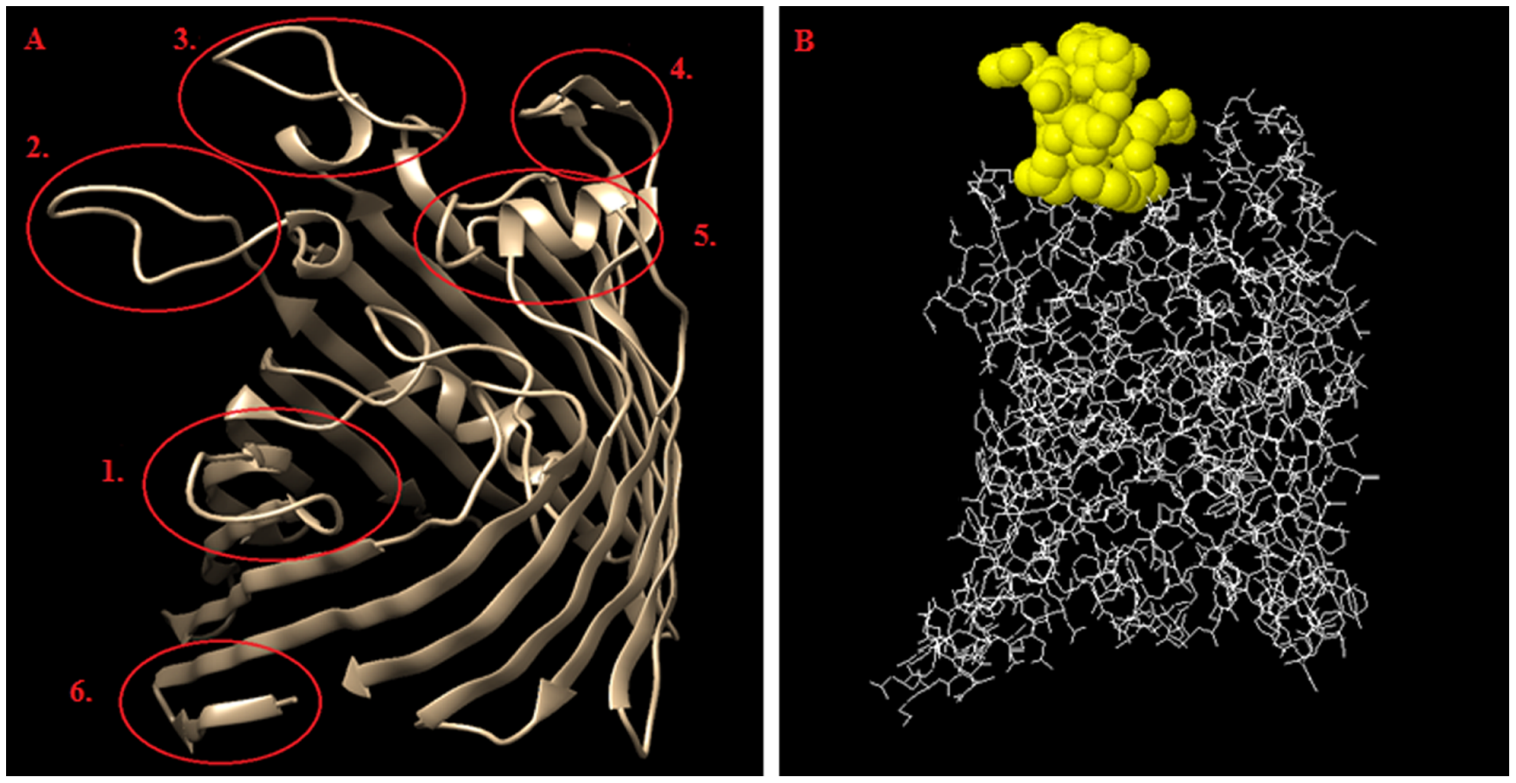
Summary of the bioinformatic studies on the OmpC. A - epitope distribution on the surface of *S. flexneri* 3a OmpC 3D model; epitopes 1 to 5 represent outer membrane loops (1– loop II, 2– loop IV, 3– loop V, 4– loop VII, 5– loop VIII) and epitope number 6 corresponds to inner-membrane loop and has been used as a control, B – location of the epitope No. 3 (RYDERY) on the OmpC surface. Images were created by using ElliPro Prediction, and edited with Chimera Software.

There is no known structure for the OmpC from *S. flexneri*. The OmpC from *S. flexneri* 3a has 94% sequence identity to OmpC from *E. coli*. Therefore, it was possible to use *E. coli* OmpC structure (PDB: 2J1N), as the template for building the structure model for the OmpC from *S. flexneri* 3a. Both *E. coli* and *S. flexneri* 3a OmpC show very high sequence homology in the β-barrel motif, while the outer membrane loops, such as loop IV, V and VII [28], are very different. As expected, these three loops obtained the highest score for their predicted antigenicity [29]–[32] (Table 3, and Fig. 2).

**Table 3:** Prediction of the discontinuous and continuous epitopes for *S. flexneri* 3a OmpC.

Predicted discontinuous epitope	Score*	Predicted continuous epitope	Score
AEVYNKDGNKLDL (1–13) **	1.35	VYNKDGNKL (1–11)**	0.43
DDKSVDGD (25–32)	1.15	YFSDDKSVDGDQT (22–34)	0.63
ETQVTDQL (43–50)**	1.16	GNSAENENNSWT (62–73)	0.82
YQIQGNSAENENNSWT (58–74)	1.36	YQGKNGSPEGEGMTNNGREALRQN (149–172)	0.84
GKNGSPEGEGMTNNGREALRQNGDGV (151–174)	1.81	SSSKRTDDQNFGLNRYDERY (194–213)	0.34
KRTDDQNFGLNRYDERYIGN (197–216)	1.56	RVGNLGWANKAQNFE (248–262)	0.13
KNLGVINGRNYDD (285–297)	1.50	KGKNLGVINGRNYDD (283–297)	0.54
DDNQFTRDAG (328–337)	1.24	NKNMS (313–317)	0.10
		LLDDNQFTRDAGINTDNI (326–343)	0.42

* the score is the average of the scores obtained for each residue.

** inner-membrane loop.

Underlined are amino acids with their side-chains exposed to the surface of the bacterial membrane and the highest probability of contributing to the antigenic epitope.

### Epitope Mapping

**Figure 3:**
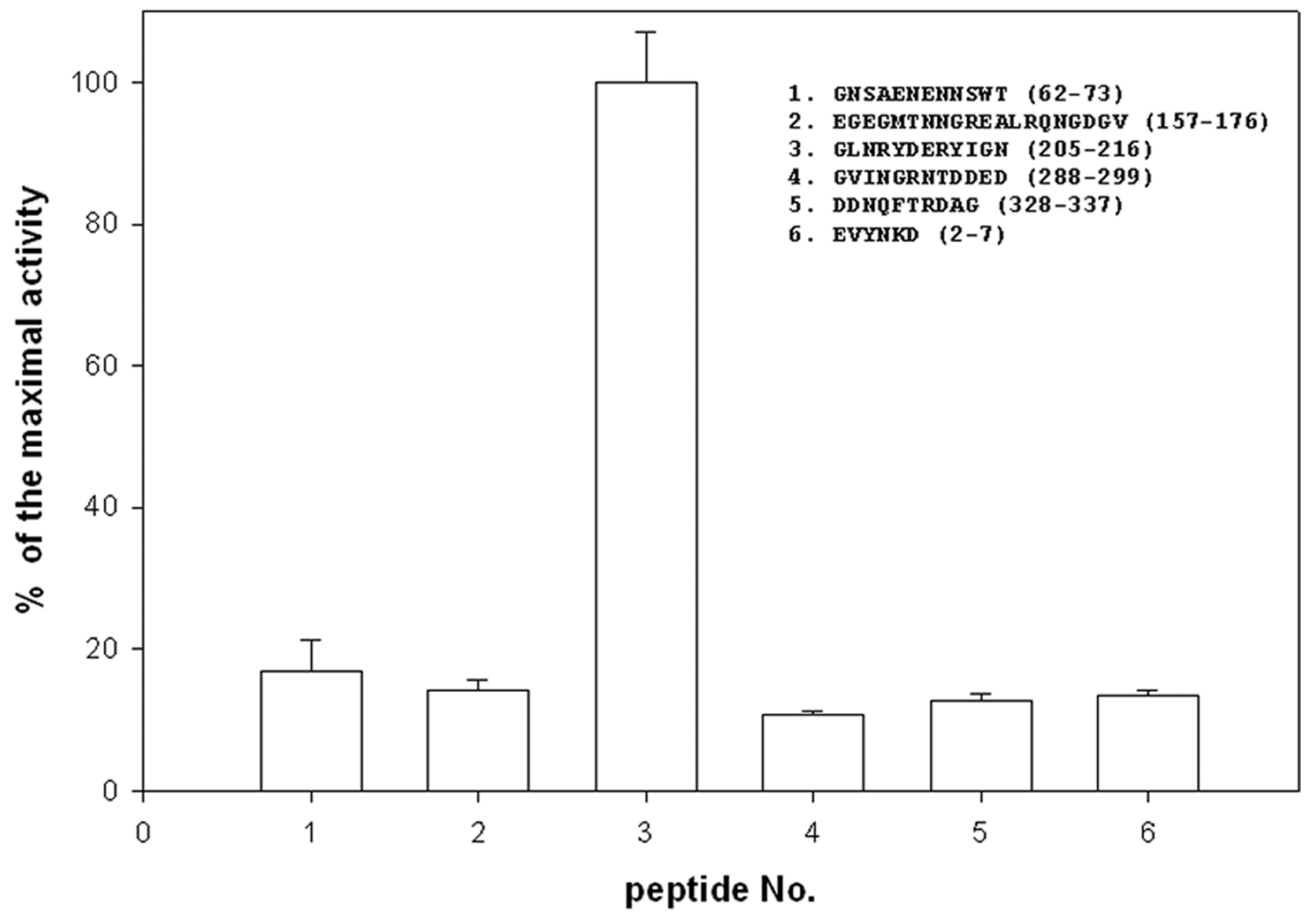
Antigenic activity of the pin-tethered peptides representing predicted epitopes. Six peptides were synthesized on polyethylene pins (MIMOTOPES, Clayton, Victoria, Australia) as the solid phase and by using the Fmoc strategy. After the synthesis peptides remained attached to the pins and were used in the ELISA assay. Unlike in a regular ELISA, formation of the [antigen]-[primary Ab] complex took place on the pin, rather than on the wall of the 96-well polystyrene plate. The primary antibody (Ab) adsorbed to the pin was detected with goat-anti-human IgG-AP conjugate, followed by reaction with orthonitrophenyl phosphate (ONPP) as the AP substrate. The IgG fraction was obtained from the mixture of thirteen umbilical cord sera. The results are the average of three independent assays.

**Figure 4:**
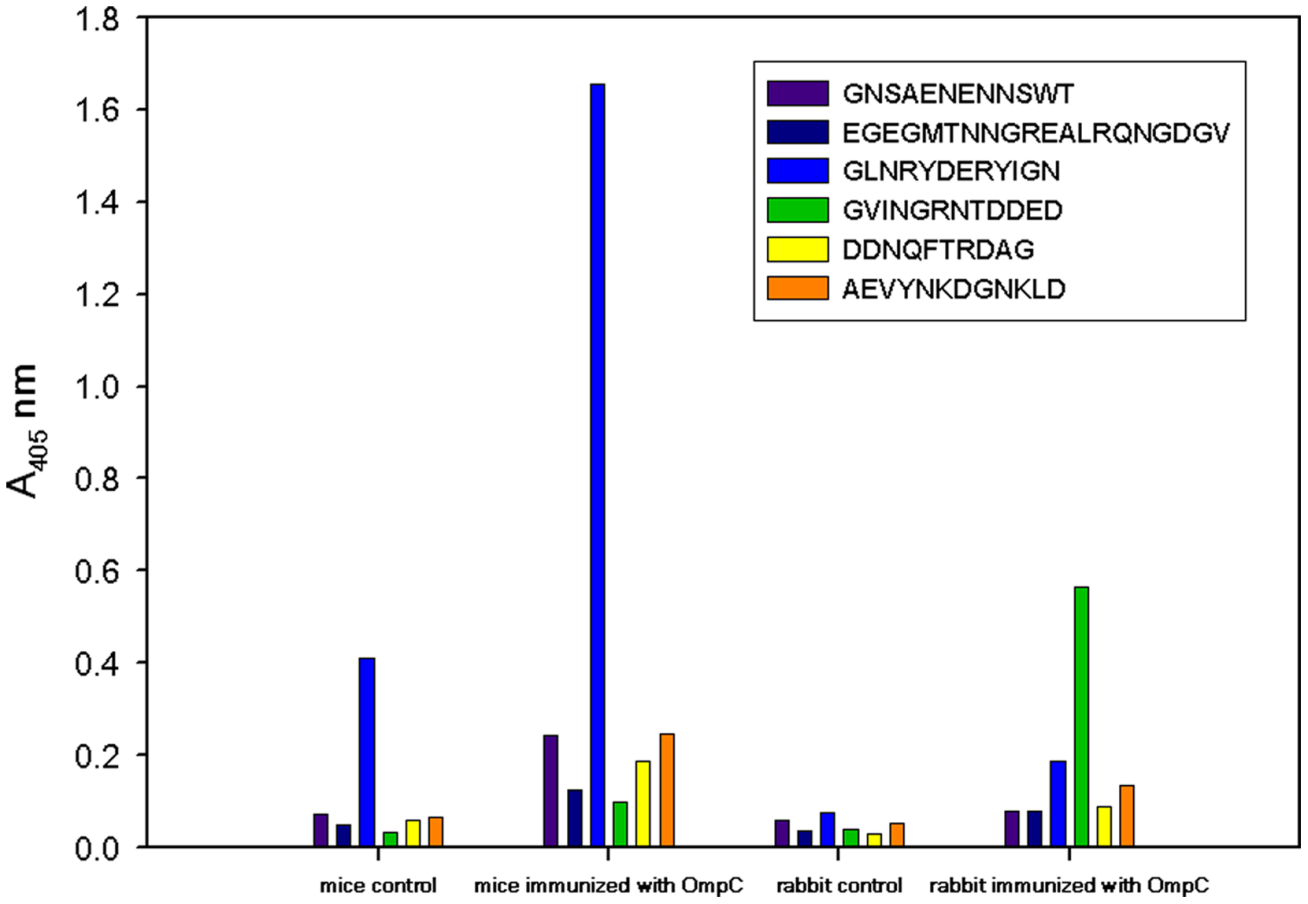
OmpC epitope recognition by mouse and rabbit sera. Mice and rabbits were immunized with purified OmpC as described in Experimental Procedures. Peptides corresponding to predicted epitopes were synthesized as described in Fig. 3. To determine immunological activity mouse and rabbit sera were used as the primary antibody (Ab) and detected with goat-anti-mouse IgG-AP conjugate, or goat-anti-rabbit IgG-AP conjugate, respectively. Sera from non-immunized animals were used as controls.

We showed earlier that IgG from umbilical cord serum binds OmpC from *S. flexneri* 3a [23], [33]. To identify which of OmpC’s projected epitopes (Table 4) are recognized by immunoglobulins, peptides bearing the sequence of the predicted epitopes were synthesized using pin technology and tested for their antigenic activity. The enzyme-linked immunosorbent assay (ELISA) results (Fig. 3) show that only GLNRYDERYIGN (peptide No. 3), spanning loop V [28], was recognized by umbilical cord serum IgG. In a separate experiment, the same six tethered peptides were allowed to react with mouse serum that came from mice that were immunized with purified *S. flexneri* 3a OmpC and survived challenge with live bacteria. Again, only peptide No. 3 was active, i.e., the same as the one reacting with human umbilical cord serum IgG. Immunization of rabbits with OmpC increased the level of antibody against peptide GVINGRNTDDED. Unlike mice, OmpC-immunized rabbits produced sera reacting with peptide 4 (Fig. 4).

**Table 4:** Synthetic epitopes and their corresponding loops in *Shigella flexneri* OmpC model.

Epitope*	Sequence	Loop**	Sequence
		I	SDDKSVD (24–30)
1	GNSAENENNSWT (62–73)	II	NSAEN (63–67)
		III	VVYDVTSWT (96–105)
2	EGEGMTNNGREALRQNGDGV (157–176)	IV	QNKGSPEGEGMTNNGREALRQNG (149–173)
3	GLNRYDERYIGN (205–216)	V	TDDQNFGLNRYDERYIGNG (199–217)
		VI	RVGNLG (248–253)
4	GVINGRNTDDED (288–299)	VII	NLGVINGRNYDD (286–297)
5	DDNQFTRDAG (328–337)	VIII	LDDNQFTRDAGINTDN (327–342)
6	AEVYNKDGNKLD (2–7)		intracellular loop, N-end

* epitopes synthesized based on the highest score from continuous and discontinuous prediction method.

** *Shigella flexneri* 3a OmpC loops assigned according to *E. coli* OmpC structure [28].

**Figure 5:**
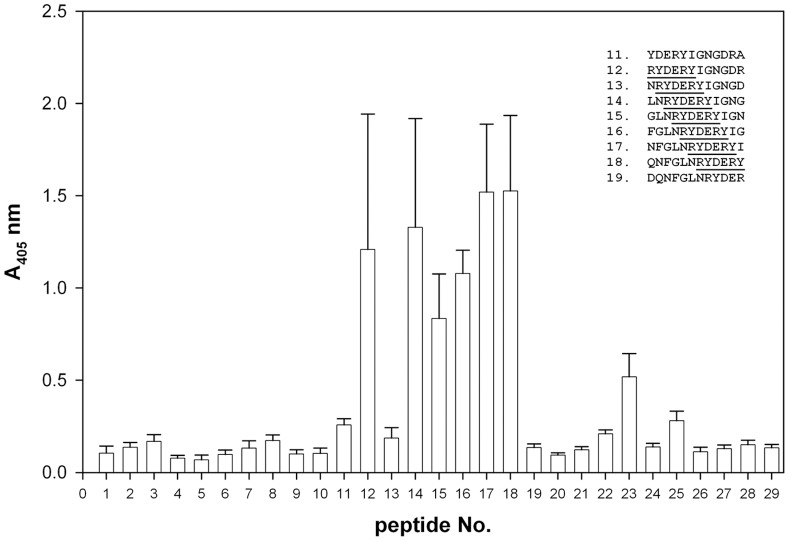
Probing loop V by using “sliding ruler” approach. Series of 29 dodecapeptides corresponding to the loop V sequence were synthesized using the pin method (Experimental Procedures). Each peptide in the series differed from its predecessor by one amino acid. The “ruler” has been moved from the C- to the N-end, allowing for complete coverage of the loop sequence. Binding of the IgG fraction from the umbilical cord serum was performed as described in Fig. 3. The results represent the average of the nine most active umbilical cord sera.

To identify the minimal length of the peptide recognized by the umbilical cord sera IgG, a library of 29 overlapping dodecapeptides was synthesized. This library covered the whole length of loop V [28] and went beyond the sequence covered by peptide No. 3 (Fig. 3). The peptides were tested for their antigenic activity (Fig. 5). Nine out of thirteen umbilical cord sera showed very high reactivity, while four sera were less reactive. The reactive sera showed the highest reactivity against peptides that contained RYDERY as part of their sequence. Therefore, recognition of peptide No. 3 of OmpC must play an important role in the immunity of newborns and their mothers.

**Figure 6:**
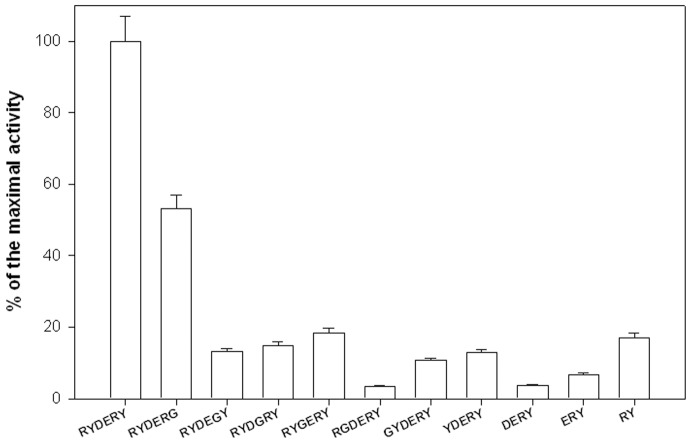
Defining epitope’s minimal sequence. Series of pin-tethered RYDERY derivatives were synthesized as described in Fig. 3. In one series individual amino acids were replaced with glycine, and in the other series amino acids were truncated one-by-one from the N-terminus. For both series antigenic activity for the peptides was determined as described in Fig. 3. The results are the average of three independent assays.

The results suggest that the RYDERY sequence defines a minimal antigenic determinant for OmpC. Detailed analysis of peptides that were either shorter than 6 amino acids or with each of the six residues systematically replaced by glycine (Fig. 6) shows that any change to the original sequence RYDERY leads to a precipitous drop in the antigenic activity. Only replacement of tyrosine-213 (Y^213^) with glycine (RYDERG) caused 50% decrease in antigenic activity. When both N- and C-terminal ends were extended by one to six amino acids, no adverse effect on the antigenic properties of the peptides was observed (data not shown). Furthermore, when glycine was used to replace one or two of the amino acids flanking either the N-, or C-terminus of the minimal antigenic determinant (e.g. GGRYDERYGG), no change was also noted in the interaction of such peptide with the umbilical cord serum IgG.

## Discussion

Subunit vaccines promise a better and more affordable method for combating diseases that negatively impact developing countries. They are also expected to surpass vaccines produced with attenuated or genetically-modified bacteria [12]–[13]. Synthetic immunogenic peptides are desirable components for subunit vaccines because unlike traditional vaccines they a) carry no infectious material, b) have no cross-reactivity with host tissues, c) can induce site-specific antibodies, d) are chemically defined and can be modified, and e) allow for swift large-scale manufacturing and long-term storage in the event of a pandemic [34].

The peptide vaccines usually are derived from bacterial surface proteins participating in host-pathogen interactions [35]–[36]. There are examples of peptide vaccines where the peptide mimics not a protein epitope, but a polysaccharide, as in the case of LPS from *S. flexneri* serotype Y [37]. Unlike vaccines derived from protein templates, the rational design of a polysaccharide-detecting vaccine is virtually impossible. On the other hand, effective peptide vaccine design requires identification of the specific epitopes within the protein antigen capable of eliciting an immunological response [38]. In this work we have demonstrated the protective immunological properties of OmpC (Table 1). The results show that immunizing mice with OmpC leads to a significant increase in the survival of mice challenged with a live bacteria. Immunized mice produced antibodies directed against loop V (peptide No. 3). Interestingly, immunization of rabbits with OmpC induced production of antibodies recognizing loop VII (peptide No. 4, Fig. 4) rather than loop V. Immunization of mice with the OmpC protein increased the existing low level of antibodies recognizing peptide No. 3 (Fig. 4). Such anti-OmpC antibodies recognizing peptide No. 3 are also present in human umbilical cord sera. Since the antibodies present in the mother’s umbilical cord blood are known to pass through the placenta to the fetus, they must protect the fetus and newborn child against the development of *Shigella* infection. This is especially important for newborns, since at the time of birth their immunological system is immature.

Due to safety concerns, it is better to vaccinate infants and older children with peptides conjugated to a carrier rather than with a vaccine containing whole attenuated bacteria. Apart from those concerns, peptide vaccines offer higher selectivity and promise better quality control, longer shelf life and an overall lower cost of manufacturing. One of the most developmentally advanced peptide vaccines, one that has already reached the final stages of clinical trials, is a vaccine against malaria [39]. The ultimate objective of our project has been to develop a peptide vaccine supporting immunity of newborns and directed against enterobacterial pathogens.

In this work we have sequenced and identified the immunogenic epitope, a part of loop V of outer membrane protein C from *S. flexneri* 3a, which was ultimately detected employing a “sliding ruler” scanning of the entire loop V. This approach allowed us to define sequence of the minimal antigenic determinant as RYDERY (Fig. 5 and Fig. 6). Such a peptide is a good candidate to become part of the anti-enterobacterial vaccine.

One of the potential adverse effects of traditional vaccines is their cross-reactivity. Such vaccines, especially when directed against *Enterobacteriaceae*, may elicit responses against commensal bacteria in the gut flora, e.g., *E. coli*. A certain physiological level of anti-commensal antibodies is necessary to maintain homeostatic balance and to prevent humoral immunodeficiency [23], while shifting that balance by eliminating the commensal strains may pave the way to secondary, often life-threatening infections. Our data suggest, that an antibody recognizing the peptide representing loop V of OmpC from *S. flexneri* 3a (Fig. 3, peptide No. 3) should cause no harm to commensal *E. coli*. It is very unlikely that such an antibody will recognize OmpC from commensal strains of *E. coli*, since there are substantial differences in the sequence of loop V. In *Shigella,* this sequence stretches between T^199^ and G^216^ (TDDQNFGLN**RYDERY**IGNG). The fragment **RYDERY** (bold) appears to be essential for the epitope’s antigenic activity. At the same time, the sequence of loop V in OmpC from the commensal strains of *E. coli* (GenBank: NC_011415, NC_011741, AEFE01000024, NC_012947, NC_012967) shows deletion of six amino acids at positions from F^204^ to Y^209^ and, instead of **RYDERY** in the critical antigenic region, it has **–TAAY** (TDAQN––**TAAY**IGNG). It must be said that the majority of *E. coli* strains produce OmpC with altered loop V. Among them are also highly pathogenic strains of *E. coli*, such as O157:H7, DEC and O55:H7, that all have the **RYDERY** motif missing from loop V. The results in Fig. 5 and 6 support the notion that an antibody recognizing loop V in OmpC from *S. flexneri* 3a will not react with OmpC from commensal and highly pathogenic strains of *E. coli*. However, OmpC is one of several enterobacterial outer membrane antigenic proteins. In our earlier work describing OMPs from several *Enterobacteriaceae* strains, namely *Citrobacter, Hafnia, Escherichia, Shigella, Klebsiella, Proteus*, *Salmonella*, we have observed recognition of these proteins by the umbilical cord sera [23]. It must be noted that some of these OMPs show a very low sequence homology to OmpC.

Synthetic peptides do not readily stimulate T cells and, because of their small size, may behave like haptens and will require coupling to a suitable protein carrier. In our future studies, we will focus on finding an optimal carrier and studying the protective properties of the antibodies induced by the conjugated peptides.

## Experimental Procedures

### Ethics Statement

The animal studies were conducted in strict accordance with the ethical guidelines established by the National Ethics Committee and approved by the First Local Ethics Commission at the Institute of Immunology and Experimental Therapy, Polish Academy of Sciences (LKE 53/2009).

Samples of the umbilical cord sera were collected from healthy women at the Obstetric Clinic of Medical University of Wroclaw. Samples were obtained with patients’ written informed consent. The use of umbilical cord sera samples in this study was approved by the Medical Ethics Committee of the Medical University of Wroclaw (KB-882-2012).

### Materials

#### Bacterial strain

*Shigella flexneri* serotype 3a strain (PCM 1793, used in other studies [23]) was obtained from the Polish Collection of Microorganisms (PCM) of the Institute of Immunology and Experimental Therapy, Polish Academy of Sciences (Wroclaw, Poland). Bacteria were grown in liquid Brain-Heart Infusion (BHI) medium (Difco) as well as in plates with enriched Bacto Agar (Difco).

#### Sera

Human umbilical cord sera from healthy women were obtained from the Obstetric Clinic of the Medical University of Wroclaw. The study was approved by the Medical Ethics Committee of the Medical University of Wroclaw. To obtain the IgG fraction, sera were precipitated to 50% of saturation with ammonium sulfate, dialyzed against PBS and dissolved to the original volume.

#### Animals

White rabbits weighing about 3 kg and six to seven-week-old mice of the inbred 129/Ao/Boy/IiW strain of both sexes weighing about 20 g were obtained from the Breeding Unit of the Medical University of Wroclaw, Poland. Animals were held in quarantine for one week before use in experiments.

### Immunological Tests

#### Immunization of animals

Rabbits were immunized subcutaneously with 2 mg of purified OmpC mixed with Freund’s adjuvant three times at two-week intervals. Exsanguination of rabbits was done ten days after the last injection. To obtain the IgG fraction, sera were precipitated to 50% of saturation with ammonium sulfate, dialyzed against PBS and dissolved to the original volume.

#### Determination of LD_100_

Groups of six mice were injected intraperitoneally with 0.2 ml of 10-fold increasing dilutions of overnight broth culture of bacteria. LD_100_ for the *Shigella flexneri* 3a strain was calculated by the method of Reed and Muench, as described elsewhere [40].

### Monophosphoryl Lipid A (MPL) Formulation

The procedure was provided for formulation of MPL, an oil-in-water emulsion, and was performed using monophosphoryl lipid A of *Hafnia alvei* PCM 1200, Squalen (Sigma), Tween 80 (Sigma) and Lecithin (Sigma) according to the method of Baldridge and Crane [41]. MPL of *Hafnia alvei* PCM 1200 used for an oil-in-water emulsion was obtained after 30 minutes of hydrolysis of lipid A in 0.1 M HCl at 100°C.

#### Protection studies

Groups of six mice were used in these studies. Mice were immunized intraperitoneally or subcutaneously once with different doses of OmpC from *S. flexneri* 3a (1.6 µg; 4.8 µg; 5 µg; 10 µg; 20 µg) or three times (1.6 µg, 3.2 µg, 1.6 µg) at 1-week intervals. Samples of the OmpC from *S. flexneri* 3a used for immunization were dissolved in PBS and mixed with MPL adjuvant in a ratio of 1∶5. One week after the last immunization the animals were challenged intraperitoneally with a lethal dose (LD_100_) of *S. flexneri* 3a. The animals were observed and their conditions as well as mortality were recorded, and the number of survivors was determined 72 h after the challenge. The control animals received only PBS or MPL adjuvant according to the same immunization schedule.

#### Titration of sera for the presence of anti-OmpC antibodies

The enzyme-linked immunosorbent assay (ELISA) was carried out according to earlier described protocols [23].

#### ELISA test with peptides on pins

The TBST (0.1% Tween 20 in Tris buffered saline) equilibrated pins were transferred to a 96-well plate filled with 200 µl of 1% BSA (bovine serum albumin) in TBST and incubated for 60 min at room temperature. After the blocking step pins were immersed in 100 µl of the IgG fraction obtained from the umbilical cord serum and incubated for 60 min at room temperature. Each IgG preparation before the assay was diluted 1∶500 with 1% BSA in TBST. Next, pins were washed three times, each time for 5 min in 10 ml of TBST. To detect bound IgG, pins were incubated with goat-anti-human IgG-alkaline phosphatase (AP) conjugate (ICN Biomedicals, Aurora, Ohio, USA) diluted according to the manufacturer’s recommendations. The level of bound conjugate is indicative of peptide antigenic activity and is measured through the AP activity. The p-nitrophenyl phosphate (pNPP Liquid substrate for ELISA, Sigma) was used to measure AP activity following the manufacturer’s protocol. Enzymatic reaction was stopped by removal of pins from the plate and released p-nitrophenol was measured at 406 nm in a plate reader (BioTek). To re-use pins they were treated with “disruption buffer” as described below.

### Isolation and Purification of *S. flexneri* 3a OmpC

*OMP extraction and OmpC purification –* A crude outer membrane proteins (OMP) fraction was extracted from lyophilized *S. flexneri* 3a bacteria with valeric acid according to Arcidiacono et al. [42]. The OmpC was further purified according to the procedure optimized in our laboratory [33]. Briefly, the crude OMP fraction was suspended in 0.4% Triton X-100 in 50 mM Tris-HCl pH 8 in 50 mM NaCl (extraction buffer) and centrifuged at 14 000×g for 30 min. The supernatant containing solubilized OMPs was loaded on a Sephacryl S-200 HR (Pharmacia) column (1.6 cm×100 cm) equilibrated with the extraction buffer. Fractions containing OmpC were pooled, dialyzed against water and concentrated by ultrafiltration (10 kDa cut-off membrane, Millipore). The concentrated sample was applied on a DE-52 HR (Whatman) column (1.6 cm×10 cm) equilibrated with 50 mM Tris-HCl pH 8 containing 10 mM EDTA and OmpC eluted with a linear gradient of 0–0.5 M NaCl. The fractions containing OmpC, as determined by immunoblotting, were collected, dialyzed against water, concentrated and stored at −20°C. Protein concentration was determined by the Lowry method [43], using BSA as the standard.

### Protein Analysis and Identification

#### 2D-SDS-PAGE

Isoelectric focusing (IEF) of proteins was done on immobilized pH gradient (IPG) strips (11 cm, pH 4–7 range, Amersham Pharmacia Biotech, Sweden). Prior to IEF protein samples were precipitated with 12.5% TCA followed by wash with acetone and dissolved in sample buffer containing 7 M urea, 2 M thiourea, 4% CHAPS and 2% DTT. The IPG strips were placed in “sacrophages” and equilibrated with 125 µl of the sample buffer containing approx. 100 µg (or less) of protein. The IEF was conducted in IPGphor (Amersham Pharmacia Biotech) at 15°C using a voltage step gradient as recommended by the manufacturer. After isoelectric focusing, the strips were equilibrated for 15 min in 10 ml of the mixture of 6 M urea, 0.5% DTT, 30% glycerol, 50 mM Tris-Cl pH 8.8, followed by 15 min incubation in 10 ml of 6 M urea, 2% iodoacetamide, 30% glycerol, 50 mM Tris-HCl pH 8.8. For the second dimension IPG strips were placed over a vertical slab of 12.5% polyacrylamide gel prepared according to Laemmli [44] and covered with 2% agarose in SDS-PAGE running buffer. The electrophoresis was run for approx. 4 h at 120 V and protein spots were visualized by non-reducing silver staining [45].

#### Identification of proteins by tryptic digestion and LC-MS/MS

Protein spots were extracted from 2D-polyacrylamide gel with an Eppendorf pipette and approx. 1 mm in diameter gel pieces were destained according to the procedure described by Gharahdaghi et al. [46]. In-gel tryptic digestion of proteins was done using Promega (Madison, WI, USA) Gold Label trypsin, following the manufacturer’s protocol with only minor alterations. Unlike in the original protocol, tryptic fragments were extracted from the polyacrylamide gel with 0.5 M urea in 90% acetonitrile and without further manipulation subjected to LC-MS/MS analysis. A tryptic digest sample (1–10 µl) was injected on a trap column coupled to a C18 Waters NanoEase capillary column 75 µm×50 mm. The ESI-MS/MS experiments were carried out on a QTOF-Global mass spectrometer (Micromass, Manchester, UK). The capillary voltage was set to an average of 3.8 kV, the sample cone to 100 V. The microchannel plate detector (MCP) was adjusted to 2050 V. The collision gas was argon with a pressure of 1 bar and collision energy was set at 25 to 40 V. Optimal operating parameters of the ESI interface were obtained by infusing standard solutions of [Glu^1^]-fibrinopeptide B, 100 fmol/µl in a solution of water: acetonitrile (50∶50 v/v) with 0.1% formic acid at a flow rate of 2.0 µl/min. Accurate instrument calibration was performed in order to achieve an error less than 4 ppm, using [Glu^1^]-fibrinopeptide B (Sigma, Oakville, ON, Canada) as the reference compound in MS/MS mode. The data were acquired using the data-dependent acquisition (DDA) method, MS/MS spectra were transformed and amino acid sequences were analyzed automatically using ProteinLynx software (Micromass).

#### OmpC cloning and DNA sequencing

The *ompC* gene from *S. flexneri* 3a was amplified by PCR from genomic DNA using the 5′ primer 5′-GGATCCGCTGAAGTGTACAAACAAAGA-3′ and the 3′ primer 5′-AAGCTTGAACTGGTAAACCAGGCCCA-3′. These primers introduce BamHI and Hind III recognition sites into the PCR product to permit directional cloning into the pQE80L vector (Qiagen, Germany). After transformation into competent *E. coli* cells (Novablue), colonies were selected for ampicillin resistance and the resultant clones were screened for the appropriate insert by agarose gel electrophoresis. The DNA sequence of the *S. flexneri* 3a *ompC* was determined at Genomed (Warsaw, Poland) and it was verified manually. The DNA sequence of the *ompC* gene from *S. flexneri* 3a was deposited in Gen Bank (Accession No. KC865276).

### Bioinformatics

#### OmpC structure model

The *Shigella flexneri* 3a OmpC structure model was obtained using the translated DNA sequence of this gene, SWISS MODEL workspace [29]–[31] and the structure of *E. coli* OmpC (PDB: 2J1N) as a template.

#### Discontinuous epitope prediction

To predict B-cell type antigenic epitopes on *Shigella flexneri* 3a OmpC, the discontinuous prediction method [32] relying on the antigen’s structural features was applied (http://pepito.proteomics.ics.uci.edu). The regions with the highest score and showing amino acid side-chains exposed to the surface of the outer membrane space were selected.

### Synthesis of Peptides Tethered to Polyethylene Pins

Peptides corresponding to predicted epitopes were synthesized using NCP Block of 96 Hydroxypropylmethacrylate pins (MIMOTOPES, Clayton, Victoria, Australia) according to the standard protocol with slight modifications [47]. This procedure was performed in 96-well plates, following the one-pin – one-peptide approach. Each pin was submersed in 100 µl of the solution containing 60 mM F-moc amino acid and an equimolar amount of diisopropylcarbodiimide and N-hydroxybenzotriazole as coupling reagents. Each coupling cycle lasted 4 h, and to assess the completion of the coupling reaction, pins were tested with 10 mM bromophenol blue for the presence of free amino groups. Finally, peptides were deprotected, but remained attached to their corresponding pins. Subsequently they were washed with MeOH, 0.5% acetic acid in MeOH, MeOH and after drying stored at −20°C. Before and after ELISA pins were immersed in the “disruption buffer” composed of 1% SDS, 0.1% 2-mercaptoethanol and 0.1 M Na_3_PO_4_, pH 7.2, heated to 60°C and placed in a sonication bath for 10 min. To remove the disruption buffer, pins were briefly rinsed with water and placed in a 60°C water- bath for 30 min. Before ELISA pins were equilibrated with TBST for 10 min at room temperature.
